# Visual and Oculomotor Function in Developmental Dyslexia: A Systematic Review and Meta-Analysis

**DOI:** 10.1007/s44402-026-00044-0

**Published:** 2026-03-09

**Authors:** Clara Martinez-Perez, Ana Paula Oliveira, Isabel Baltazar

**Affiliations:** 1 Applied Physics Department (Optometry Area), Facultade de Óptica e Optometría, Universidade de Santiago de Compostela, Santiago de Compostela, Spain; 2 Instituto Superior de Educação e Ciências de Lisboa (ISEC Lisboa), Lisboa, Portugal; 3 Centro de Investigação, Desenvolvimento e Inovação em Turismo (CiTUR) – Polo Estoril, Estoril, Portugal

**Keywords:** Binocular vision, Dyslexia, Eye movements, Oculomotor function, Saccades, Vergence

## Abstract

**Topic:**

To determine whether individuals with developmental dyslexia present differences in visual and oculomotor functions compared with age-matched controls.

**Clinical Relevance:**

Developmental dyslexia affects a substantial proportion of school-aged children, with prevalence estimates ranging between 3% and 6%, depending on diagnostic criteria. It is characterised by persistent reading difficulties despite normal intelligence and education. Although phonological deficits are well established, the contribution of visual and oculomotor anomalies remains debated. Identifying consistent visual differences may support more comprehensive assessments and targeted interventions alongside educational strategies.

**Methods:**

This systematic review and meta-analysis, registered in PROSPERO (CRD420251119429), included observational case-control studies comparing visual and oculomotor functions in individuals with developmental dyslexia and age-matched controls. Searches were conducted in PubMed, Web of Science, and Scopus. Outcomes included binocular vision, oculomotor performance, accommodation, visual acuity, refractive error and contrast sensitivity. Methodological quality was assessed using the MINORS tool and certainty of evidence using GRADE.

**Results:**

Twenty-six studies with 8 to 124 participants per group were included. Dyslexic individuals showed significantly greater near exophoria (mean difference 0.84 prism diopters, 95% CI: 0.22 to 1.46) and reduced near fusional vergence ranges, including negative (–6.42 prism diopters, 95% CI: –8.65 to –4.19) and positive fusional vergence (–6.72 prism diopters, 95% CI: –8.66 to –4.77), all *p* < 0.01. Oculomotor differences included a higher number of fixations, longer fixation duration, more regressions and reduced saccade amplitude. No significant group differences were found for refractive error or visual acuity.

**Conclusions:**

Children with developmental dyslexia exhibit consistent binocular and oculomotor anomalies that may increase visual effort during reading. Incorporating targeted assessment of these functions into vision care may complement multidisciplinary management. Further research is needed to clarify their clinical relevance.

Key Points

- Children with developmental dyslexia show consistent differences in eye movement control and eye coordination, indicating that visual factors may contribute to reading difficulties in a subset of affected individuals.
- Basic measures of eyesight, such as clarity of vision and focusing accuracy, do not differ between dyslexic and typical readers, confirming that dyslexia cannot be explained by simple visual or optical problems.
- Including assessment of eye movement efficiency and eye coordination in clinical evaluations may help identify visual contributors to reading effort and support more comprehensive approaches to dyslexia management.

## Introduction

Vision and its associated oculomotor functions are fundamental to many aspects of daily life, with reading standing out as one of the most visually and cognitively demanding tasks, especially in childhood, when both visual and neurological development are ongoing [1]. Saccadic eye movements (rapid, coordinated shifts in fixation) are critical for fluent reading, allowing efficient processing of text by bringing new words or symbols onto the fovea [2, 3]. The precision of these movements depends on both ocular motor control and a complex interplay with attentional and cognitive systems [1]. This coordination develops throughout childhood, mirroring improvements in reading skills and frontal cortex maturation [2, 4].

Developmental dyslexia is a common neurodevelopmental disorder, with population-based studies reporting prevalence estimates generally ranging between approximately 3% and 6% in school-aged children, depending on diagnostic criteria and assessment methods [5–8]. Higher prevalence figures are often reported when learning disabilities are considered as a broader category rather than developmental dyslexia specifically. Characterised by persistent difficulty in learning to read despite normal intelligence and schooling [1, 9], dyslexia is typically linked to phonological deficits, challenges in mapping letters to sounds that lead to slow reading, poor comprehension and frequent spelling errors [10, 11]. However, visual and oculomotor anomalies are also frequently reported, prompting ongoing debate as to whether they are a consequence or a contributing factor in dyslexia [9, 12].

In this context, neurocognitive models emphasise that developmental dyslexia is not a unitary disorder but a heterogeneous condition in which distinct cognitive deficits may differentially contribute to similar reading impairments. Neuroimaging evidence from Danelli et al. [13] demonstrates that phonological, visual magnocellular and motor/cerebellar systems converge on partially overlapping yet functionally distinct neural substrates, supporting the notion that multiple pathways can lead to comparable reading difficulties. Importantly, not all individuals with developmental dyslexia exhibit the same pattern of cognitive or visual deficits. Neuroimaging and cognitive evidence indicate that phonological, visual magnocellular and motor/oculomotor impairments may differentially contribute to reading difficulties across individuals, rather than constituting a single core deficit. As demonstrated by Danelli et al. [13], partially overlapping but functionally distinct neural systems can converge on similar reading impairments, supporting the existence of heterogeneous profiles and potential subtypes of dyslexia. This heterogeneity has important implications for both the interpretation of group-level findings and the development of individualised assessment and intervention strategies.

Studies have consistently found that children with dyslexia show abnormal eye movement patterns during reading, including more fixations and regressions, longer fixation durations and increased frequency of disconjugate saccades compared to controls [11, 14–18]. Some of these issues, such as higher vergence errors, are thought to stem from poor binocular coordination of saccades, leading to greater visual effort and transient diplopia [11, 14–16]. Thus, effective reading depends not only on accurate saccades but also on precise binocular coordination, supported by adaptive interactions between saccades and vergence [14, 16, 19]. Experimental data indicate that artificially disrupting the vergence-accommodation relationship with prisms or lenses can worsen saccadic disconjugacy, highlighting the importance of integrated visual control [20].

One influential framework is the magnocellular theory, which posits that dysfunction in the magnocellular visual pathway, critical for motion detection, fixation stability and saccades, may underlie some visual and phonological symptoms of dyslexia [21, 22]. Still, neuroimaging studies suggest a bidirectional relationship: interventions that improve reading skills can also normalise activity in visual motion areas such as V5/MT [23–25], supporting a complex, reciprocal relationship between vision and reading.

Beyond oculomotor function, binocular vision parameters were also evaluated, including vergence function (convergence and divergence) and fusional vergence ranges [9]. The prevalence of visual anomalies among dyslexic children varies widely, from less than 20% to nearly 80%, depending on the study and population [11, 26]. While many studies report normal visual acuity and stereoacuity in dyslexic children [16, 27], others find higher rates of accommodative insufficiency, convergence insufficiency or reduced fusional vergence [28–30].

These findings matter to eye care practitioners, as undetected visual anomalies, even if not the primary cause of dyslexia, may worsen reading difficulties and impact academic achievement [9, 31]. There is also a high degree of comorbidity between dyslexia and visual dysfunction, which supports the need for careful visual assessment in all children with reading problems [32].

Ophthalmic assessment in dyslexia often includes the Developmental Eye Movement (DEM) test, which evaluates saccadic function through rapid number naming [33–35]. As highlighted in the comprehensive review by Facchin et al. [36], the DEM test is a simple and widely used psychometric tool with available normative data across multiple languages and populations, and it has demonstrated clinical utility in identifying vision-related reading difficulties. However, the DEM primarily captures a functional aspect of eye movements associated with reading performance rather than providing a direct, objective measurement of oculomotor parameters. While DEM scores correlate with reading performance and help distinguish oculomotor from phonological deficits, their ability to quantify eye movement quality directly is debated [37, 38], and few studies combine DEM with objective eye tracking, especially in dyslexic children [39].

The link between vision and dyslexia remains controversial in the literature. Major societies, including the American Academy of Pediatrics and American Academy of Ophthalmology, state that current evidence does not support a causal role for mild visual anomalies in learning disabilities such as dyslexia and caution against therapies without proven benefit [40]. However, many optometric authorities believe that correcting visual problems can ease reading for affected children, even if vision is not the root cause [32, 41, 42].

Given the heterogeneity in published data and ongoing clinical uncertainty, high-quality evidence synthesis is needed to clarify the extent, nature and significance of visual dysfunction in dyslexia. To address this gap, the present review provides the first meta-analysis to quantify and compare a broad range of visual and oculomotor outcomes systematically between individuals with dyslexia and controls. The findings aim to clarify the clinical relevance of these differences for vision science and to inform multidisciplinary management of reading disorders.

## Methods

### Research Question and PICOS Framework

**Fig. 1:**
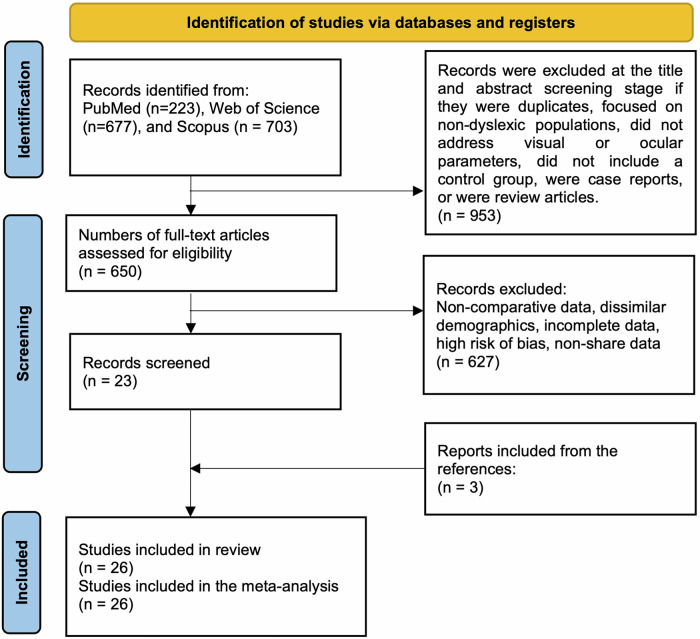
PRISMA (Preferred Reporting Items for Systematic Reviews and Meta-Analyses) flow diagram of study selection.

This systematic review and meta-analysis were registered in PROSPERO (International Prospective Register of Systematic Reviews; registration number: CRD420251119429) and conducted according to the Preferred Reporting Items for Systematic Reviews and Meta-Analyses (PRISMA) guidelines [43] and the AMSTAR-2 (A Measurement Tool to Assess Systematic Reviews) methodological standards [44] (see Fig. 1). A completed PRISMA 2020 checklist is provided as Supplementary Material (Additional file 1). The last literature search was completed on 28 July 2025.

The research question was formulated using the PICOS framework to ensure methodological rigour and relevance. Specifically, it aimed to determine whether children, adolescents and adults diagnosed with developmental dyslexia (Population) present significant differences in visual and ocular function parameters (Outcomes) compared to age-matched individuals without dyslexia (Comparator). All included studies were observational case-control designs (Study design), and the exposures of interest were a wide range of visual and ocular function assessments (Intervention/Exposure), including measures of binocular vision (such as stereopsis, near point of convergence (NPC), heterophoria and fusional vergence), oculomotor performance (saccades, fixations, regressions, latency, velocity, disconjugacy), accommodative function, visual acuity, refractive error and contrast sensitivity.

Primary outcomes focused on quantifying differences between dyslexic and control groups in these visual and oculomotor domains. Secondary analyses explored the influence of demographic variables, diagnostic approaches and methodological heterogeneity across studies. Through this comprehensive approach, the present review sought to synthesise the available evidence on visual function in dyslexia, highlight areas of consensus and divergence and identify priorities for future research.

### Eligibility Criteria

Studies were included if they met the following criteria: an observational case-control design; participants diagnosed with developmental dyslexia based on clearly defined diagnostic criteria and an age-matched control group without dyslexia; assessment of at least one visual, binocular, accommodative or oculomotor outcome, including measures of binocular vision, oculomotor performance, accommodative function, visual acuity, refractive error or contrast sensitivity and availability of sufficient quantitative data to allow effect size calculation. Studies were excluded if they were case reports, case series or lacked a control group; systematic or narrative reviews; duplicate publications derived from the same dataset or if they demonstrated insufficient methodological rigour, defined as a very low methodological quality rating according to the Methodological Index for Non-Randomized Studies (MINORS) criteria. Additional exclusion criteria included non-comparable or incomplete demographic data, unclear or inadequate diagnostic criteria for dyslexia, absence of relevant visual or oculomotor outcomes or data that could not be quantitatively compared or pooled for meta-analytic synthesis, such as missing means and standard deviations.

### Information Sources

A comprehensive and systematic literature search was performed using three major electronic databases, PubMed, Web of Science and Scopus, without restrictions on publication date or language. To ensure thorough coverage, reference lists of all included articles were screened manually to identify additional relevant studies that may have been missed in the initial database search.

### Search Methods for Identification of Studies

The search strategy combined controlled vocabulary and free-text terms related to dyslexia and visual function, including: (‘dyslexia’ OR ‘dyslexic’) AND (‘saccadic movement’ OR ‘saccades’ OR ‘stereopsis’ OR ‘ocular motility’ OR ‘vergence dysfunction’ OR ‘oculomotor’ OR ‘oculomotor dysfunction’ OR ‘contrast sensitivity’ OR ‘refractive errors’ OR ‘binocular vision’ OR ‘convergence insufficiency’ OR ‘accommodation disorder’). Complete search strategies for each database are provided in Additional file 2. Two reviewers independently assessed study eligibility at both the title/abstract screening and full-text review stages. Disagreements were resolved through discussion and consensus. No language restrictions were applied and studies published in languages other than English were translated and included when relevant data were available.

### Data Extraction and Data Items

Two authors (APO and CMP) independently extracted data from all eligible studies. For each included study, key characteristics were collected, such as first author’s name, year of publication, country or region, study design, sample size for dyslexic and control groups, mean age of participants, diagnostic criteria for dyslexia and the specific visual or oculomotor parameters assessed. Discrepancies in data extraction or study inclusion were resolved through discussion and consensus, without the need for a third reviewer. Record management, including duplicate removal and tracking of study eligibility, was conducted using Microsoft Excel (microsoft.com).

The primary variables extracted included measures of binocular vision (e.g., stereopsis, NPC, heterophoria, fusional vergence), oculomotor performance (number and duration of fixations, saccades, regressions, saccade amplitude, latency, velocity, disconjugacy), accommodative function, visual acuity, refractive error and contrast sensitivity. Additional variables, such as country/region, age range, diagnostic methods for dyslexia and reporting of confounding factors (e.g., exclusion of neurological or ophthalmic comorbidities), were also recorded to support subgroup analyses and assessment of methodological heterogeneity.

### Risk of Bias Assessment

The methodological quality and risk of bias of the included observational studies were assessed independently by two reviewers using the MINORS developed by Slim et al. [45] (see Table 1). The MINORS tool evaluates key aspects of study design, including the clarity of study objectives, consecutive inclusion of participants, appropriateness of inclusion criteria, objectivity of outcome assessments and adequacy of follow-up.

**Table 1:** Assessment of the quality of studies through Methodological Index for Non-Randomized Studies (MINORS).

Study	Clearly stated aim	Consecutive patients	Prospective collection of data	Endpoints	Assessment endpoint	Follow-up period	Loss less than 5%	Study size	Adequate control group	Contemporary group	Baseline control	Statistical analyses	MINORS
Barela et al. [48]	2	2	2	2	2	2	2	1	2	2	2	2	23
Bonifacci et al. [49]	2	2	1	2	2	2	2	1	2	2	2	2	22
Brenk-Krakowska et al. [50]	2	2	1	2	2	2	2	1	2	2	2	2	22
Bucci et al. [51]	2	2	1	2	2	2	2	1	2	2	2	2	22
Bucci et al. [18]	2	2	2	2	2	2	2	0	2	2	2	2	22
Bucci et al. [30]	2	2	2	2	2	2	2	0	2	2	2	2	22
Cornelissen et al. [52]	2	1	2	2	2	2	2	0	2	2	2	2	21
Darvishi et al. [53]	2	2	2	2	2	2	2	1	2	2	2	2	23
De Luca et al. [54]	2	2	2	2	2	2	2	1	2	2	2	2	23
Feizabadi et al. [55]	2	2	2	2	2	2	2	1	2	2	2	2	23
Hawelka et al. [56]	2	2	2	2	2	2	2	1	2	2	2	2	23
Huang et al. [57]	2	2	2	2	2	2	2	0	2	2	2	2	22
Jafarlou et al. [58]	2	2	2	2	2	2	2	0	2	2	2	2	22
Jainta and Kapoula [14]	2	2	2	2	2	2	2	0	2	2	2	2	22
Moiroud et al. [39]	2	2	2	2	2	2	2	0	2	2	2	2	22
Mukhtar et al. [59]	2	2	2	2	2	2	2	2	2	2	2	2	24
Pan et al. [60]	2	1	2	2	2	2	2	1	2	2	2	2	22
Quercia et al. [61]	2	2	2	2	1	2	2	0	2	2	2	2	21
Razuk et al. [62]	2	2	2	2	2	2	2	0	2	2	2	2	22
Seassau et al. [15]	2	2	2	2	2	2	2	0	2	2	2	2	22
Tiadi et al. [63]	2	2	2	2	2	2	2	0	2	2	2	2	22
Trauzettel-Klosinski et al. [64]	2	2	2	2	2	2	2	0	2	2	2	2	22
Vagge et al. [65]	2	2	2	2	2	2	2	0	2	2	2	2	22
Vilhena et al. [66]	2	1	2	2	2	2	2	0	2	2	2	2	21
Ward and Kapoula [67]	2	1	2	2	2	2	2	0	2	2	2	2	21
Ward and Kapoula [12]	2	1	2	2	2	2	2	0	2	2	2	2	21

For comparative studies (case-control design), the total MINORS score ranges from 0 to 24, with studies classified as very low quality (0–6), low quality (7–10), moderate quality (11–15) or high quality (16–24). Although the MINORS instrument also provides a scoring system for non-comparative studies, only comparative (case-control) studies were included in this review. Any disagreements in quality assessment were resolved through discussion until consensus was reached.

### Assessment of Results

For continuous outcomes measured on the same scale, mean differences (MD) with 95% confidence intervals (CI) were calculated. When outcomes were reported using different measurement scales, standardised mean differences were used to facilitate comparability across studies. For dichotomous outcomes, odds ratios (OR) with 95% CI were computed. Statistical heterogeneity among studies was assessed using the *I*² statistic and interpreted as low (<25%), moderate (25–50%) or high (>50%) heterogeneity. A fixed-effects model was applied when heterogeneity was not significant (*I*² ≤ 50%), while a random-effects model was used when heterogeneity was substantial. Missing or incomplete data were addressed in accordance with methodological recommendations outlined in the Cochrane Handbook for Systematic Reviews of Interventions [46]. All statistical analyses and figure generation were performed using Review Manager (RevMan) version 5.4.1 (revman.cochrane.org).

### Publication Bias

Potential publication bias was assessed through visual inspection of funnel plots generated using Review Manager (RevMan) version 5.4.1. Asymmetry in the funnel plot was interpreted as a possible indication of publication bias, reflecting the potential non-publication of smaller studies with null or inconclusive results.

### Additional Analyses

Sensitivity analyses were performed to evaluate the robustness of the pooled results by sequentially removing studies identified as highly influential within each outcome domain. These analyses allowed assessment of the impact of individual studies on overall effect estimates and contributed to a better understanding of sources of heterogeneity, particularly for key outcomes such as oculomotor parameters, binocular vision and visual acuity. All analyses were conducted using Review Manager (RevMan) version 5.4.1, applying a random-effects model when significant heterogeneity was present. Furthermore, the certainty of evidence for each outcome was evaluated using the GRADE (Grading of Recommendations, Assessment, Development and Evaluation) approach, considering factors such as risk of bias, inconsistency among studies, imprecision of effect estimates and potential publication bias [47]. All assessments were performed independently by two reviewers. Discrepancies were addressed through discussion and when necessary, a third author was consulted to reach consensus.

### Ethics Statement

This study did not meet the criteria for human subjects research as defined by our institution, as it did not include patient data. Therefore, it did not require institutional review board approval or informed consent. The study adhered to the Declaration of Helsinki.

## Results

### Study Selection

A total of 1603 records were initially retrieved from PubMed (*n* = 223), Web of Science (*n* = 677) and Scopus (*n* = 703) (Fig. 1). After removal of duplicates and screening of titles and abstracts, 953 records were excluded. Exclusion criteria at this stage included studies focused on non-dyslexic populations, those not addressing visual or ocular parameters, lack of a control group, case reports or review articles. Subsequently, 650 full-text articles were assessed for eligibility. Of these, 627 were excluded due to non-comparative data, dissimilar demographic characteristics, incomplete data, high risk of bias or unavailability of shared data. Additionally, two relevant studies were identified through manual review of reference lists. In total, 26 studies met the inclusion criteria and were included in the qualitative synthesis and meta-analysis: Barela et al. [48], Bonifacci et al. [49], Brenk-Krakowska et al. [50], Bucci et al. [51], Bucci et al. [18], Bucci et al. [30], Cornelissen et al. [52], Darvishi et al. [53], De Luca et al. [54], Feizabadi et al. [55], Hawelka et al. [56], Huang et al. [57], Jafarlou et al. [58], Jainta and Kapoula [14], Moiroud et al. [39], Mukhtar et al. [59], Pan et al. [60], Quercia et al. [61], Razuk et al. [62], Seassau et al. [15], Tiadi et al. [63], Trauzettel-Klosinski et al. [64], Vagge et al. [65], Vilhena et al. [66], Ward and Kapoula [67] and Ward and Kapoula [12].

### Study Characteristics

Table 2 summarises the key characteristics of the 26 observational case-control studies included in this meta-analysis, all of which investigated ocular and visual function parameters in individuals with dyslexia compared to age-matched controls. The studies were conducted across diverse geographic regions, including Brazil, Poland, Italy, France, the United Kingdom, Iran, Austria, China, Nigeria, Germany and Portugal. Sample sizes ranged from 14 to 124 participants, with mean ages spanning from approximately 8 to 22 years. All studies employed non-randomised, observational, case-control designs and investigated a broad array of visual functions. These included oculomotor parameters (such as saccades, fixations and pursuit movements), binocular coordination (fixation disparity, fusional vergence, heterophoria and convergence), accommodative function, stereopsis, visual acuity, contrast sensitivity and eye movement dynamics during reading tasks. Most studies used standardised diagnostic criteria for dyslexia, including international diagnostic batteries (such as the Batterie Analytique du Langage Écrit (L2MA), the Dyslexia and Dysorthography Evaluation battery (DDE-2), the MT Reading Test and Diagnostic and Statistical Manual of Mental Disorders–based protocols) and assessments by multidisciplinary teams or certified specialists. None of the included studies declared conflicts of interest.

**Table 2:** Baseline characteristics of the 26 included studies.

Author	Region	Study design	Sample size (dyslexia/control)	Mean age (years unless stated otherwise) (dyslexia/control)	Binocular vision parameters assessed	Dyslexia diagnostic criteria	COI
Barela et al. [48]	Brazil	Observational	12/12	10.8 ± 1.1; 10.4 ± 1.5	Oculomotor function (saccades, fixations)	Diagnosis at Brazilian Dyslexic Association; neurological, psychological and phonological assessment; age-matched controls	No
Bonifacci et al. [49]	Italy	Observational	18/42	125.7 ± 5.5/125.6 ± 4.6 months	Eye movement during reading (saccades, fixations, regressions)	Clinical diagnosis following Italian criteria: <−2 SD in ≥2 of 6 parameters (words, non-words, texts; speed and accuracy)	No
Brenk-Krakowska et al. [50]	Poland	Observational	25/25	21.9 ± 1.6/22.9 ± 2.5	Binocular coordination (fixation disparity, fusional vergence)	Documented developmental dyslexia diagnosed by psychologists based on significant discrepancies between literacy skills and cognitive abilities	No
Bucci et al. [51]	France	Observational	16/14	11.1 ± 1.1/12.1 ± 1.0	Oculomotor and vergence function (saccades, vergence, latency)	L2MA battery (Chevrie-Muller), IQ normal (WISC-III)	No
Bucci et al. [18]	France	Observational	12/19	11.0 ± 0.6; 11.0 ± 0.9	Oculomotor function (saccade amplitude, disconjugacy, fixations)	L2MA battery (>2 SD), WISC-IV IQ 80–115, normal stereo (≤55″ TNO)	No
Bucci et al. [30]	France	Observational	30/30	9.8 ± 0.28; 9.9 ± 0.35	Oculomotor function (fixations, pursuits, pro-/anti-saccades)	L2MA battery (≥2 SD), WISC-IV IQ 85–115, ADHD and DCD excluded, VA ≥ 20/20, normal stereoacuity (TNO), NPC ≤ 5 cm	No
Cornelissen et al. [52]	U.K.	Observational	29/29	116.7 ± 14.2/118.7 ± 8.4 months	Contrast sensitivity, motion detection	Reading age ≥2 SD below IQ-predicted level (British Ability Scales); IQ > 70; adequate schooling; clinical history of unexpected reading failure	No
Darvishi et al. [53]	Iran	Observational	32/32	8.1 ± 0.8/8.1 ± 0.8	Visual function (visual acuity, refraction, accommodation, stereoacuity, contrast sensitivity)	Evaluation by professional team (psychiatrists and speech therapists) using globally accepted diagnostic approaches; IQ ≥ 90 (Stanford–Binet)	No
De Luca et al. [54]	Italy	Observational	16/16	11.9 [11.3–12.9]/11.6 [11.1–13.4]	Eye movement during reading (fixations, saccades, regressions, silent pauses)	≥1.65 SD below the mean in speed or accuracy on the MT Reading Test	No
Feizabadi et al. [55]	Iran	Observational	27/40	8.85 ± 1.51/9.02 ± 1.34	Accommodation and binocular function (NPA, NPC, stereopsis)	Clinical diagnosis by learning specialist, normal IQ (WISC/Stanford–Binet), 7–13 years, no strabismus/amblyopia, VA < 10/10, no neurological pathology	No
Hawelka et al. [56]	Austria	Observational	18/18	17.67 ± 1.25/17.50 ± 1.08	Eye movement during reading (gaze, fixations, saccades, regressions)	Reading speed <10th percentile on standardised German sentence-reading test; IQ > 90 (WAIS-R); history of severe persistent reading speed deficit	No
Huang et al. [57]	China	Observational	28/28	10.12 ± 1.42/10.06 ± 1.29	Eye movement during reading (fixation duration, saccades, fixations)	ICD-10 criteria; PRS < 60; IQ > 70 (WISC); poor academic performance; exclusion of ADHD and other disorders	No
Jafarlou et al. [58]	Iran	Observational	30/20	9.1 ± 1.2/9.6 ± 0.9	Oculomotor function (pursuit, optokinetic, saccade latency, velocity, nystagmus)	DSM-V diagnosis by multidisciplinary team in reading disorder centres	No
Jainta and Kapoula [14]	France	Observational	13/7	11.7 ± 2/12.7 ± 1	Binocular coordination (disconjugacy, fixation disparity)	L2MA battery (>2 SD), normal IQ, no neurological/ocular pathology	No
Moiroud et al. [39]	France	Observational	13/13	10.4 ± 0.43/10.3 ± 0.46	Eye movement and binocular function (fixations, saccades, convergence/divergence, NPC, stereopsis)	L2MA ≤−2 SD, IQ 80–115 (WISC-IV), normal VA, binocular vision, no ADHD, no ocular/neurological pathology	No
Mukhtar et al. [59]	Northern Nigeria	Observational	22 / 22	12 ± 2/12 ± 2	Visual function (acuity, refraction, accommodation, facility, stereopsis, heterophoria, NPC, vergence, oculomotor skills)	Diagnosed by educational psychologist; exclusion of neurological/mental disorders or previous dyslexia interventions	No
Pan et al. [60]	China	Observational	30 / 26	10.7 ± 0.3; 10.6 ± 0.4	Eye movement during rapid automatised naming (gaze, saccades, fixations, landing positions)	Character recognition ≥1.5 SD below grade mean; C-WISC IQ ≥ 85; normal/corrected VA; Mandarin native speakers	No
Quercia et al. [61]	France/Italy	Observational	14 / 10	10.7 ± 1.2; 11.3 ± 1.6	Visual scotomas, binocular coordination (heterophoria, convergence, stereopsis)	Documented diagnosis; ≥24mo literacy delay; Odedys2 classification; exclusion of strabismus, amblyopia, etc.	No
Razuk et al. [62]	France/Brazil	Observational	18 / 18	9.8 ± 1.2 (both groups)	Eye movement and binocular function (fixations, saccades, regressions, convergence/divergence, stereopsis, NPC)	L2MA battery: ≥2 SD below mean; IQ normal; complete dyslexia evaluation; exclusion of ophthalmologic disorders	No
Seassau et al. [15]	France	Observational	43/42	10.6 ± 1.6/10.7 ± 1.5	Oculomotor function (fixations, saccades, disconjugacy)	L2MA battery (≥2 SD below mean), WISC-IV IQ 80–115; normal VA, stereoacuity, NPC; no ADHD, no neurological/psychiatric disorders	No
Tiadi et al. [63]	France	Observational	55/55	10.1 ± 0.2/10.4 ± 0.3	Oculomotor function (number of saccades)	Diagnosis with L2MA battery (≥2 SD), normal IQ (WISC-IV 85–115), ADHD excluded, normal VA/binocular vision	No
Trauzettel-Klosinski et al. [64]	Germany	Observational	16/16	9.5 ± 0.3/9.6 ± 0.4	Eye movement during reading (saccades, regressions, fixations, reading speed)	Performance ≤16th percentile and ≥1.5 SD below expected on German standardised reading tests	No
Vagge et al. [65]	Italy	Observational	11/11	9.4 ± 1.6; 9.2 ± 1.5	Oculomotor function during reading (fixation stability, saccades, regressions)	DSM-IV developmental dyslexia; DDE-2 battery (reading accuracy ≥2 SD below norm); IQ > 85; normal VA/orthoptic exam	No
Vilhena et al. [66]	Brazil/Portugal	Observational	62/62	13.8 ± 3.9;13.8 ± 4.4	Eye movement during reading (fixations, regressions, span, rate, efficiency)	Formal DSM-5 DD diagnosis; VA ≥ 20/20; text comprehension ≥60%; exclusion of ADHD and other disorders	No
Ward and Kapoula [67]	France	Observational	47/44	15.4;14.8	Oculomotor and vergence function (saccades, vergence, disconjugacy, binocular coordination)	Diagnosed in multidisciplinary centres; severe DD; specialised dyslexia schools; exclusion of neurological/psychiatric disorders	No
Ward and Kapoula [12]	France	Observational	47/44	15.4;14.8	Oculomotor function during reading (saccade amplitude, velocity, disconjugacy, fixation duration, regressions, WPM, errors)	Multidisciplinary diagnosis in specialised centres; DSM-based criteria	No

*ADHD* attention deficit hyperactivity disorder, *CI* cognitive impairment, *COI* conflict of interest, *DCD* developmental coordination disorder, *DEM* Developmental Eye Movement test, *DSM* Diagnostic and Statistical Manual of Mental Disorders, *DDE-2* Batteria per la Diagnosi della Dislessia e Disortografia Evolutiva, *IQ* intelligence quotient, *L2MA* L’Analyse des Lectures et des Liaisons de Mots, *MEM* monocular estimate method (dynamic retinoscopy), *MO* months, *MT Reading Test* Prova di Lettura MT, *NPC* near point of convergence, *NPA* near point of accommodation, *PRS* Pupil Reading Score, *RAN* rapid automatised naming, *SD* standard deviation, *TNO* Netherlands Organisation for Applied Scientific Research stereo test, *SLRT, ZLT, WLLP* German standardised reading tests, *VA* visual acuity, *WISC* Wechsler Intelligence Scale for Children, *WAIS-R* Wechsler Adult Intelligence Scale–Revised, *WPM* words per minute.

### Outcomes

**Fig. 2:**
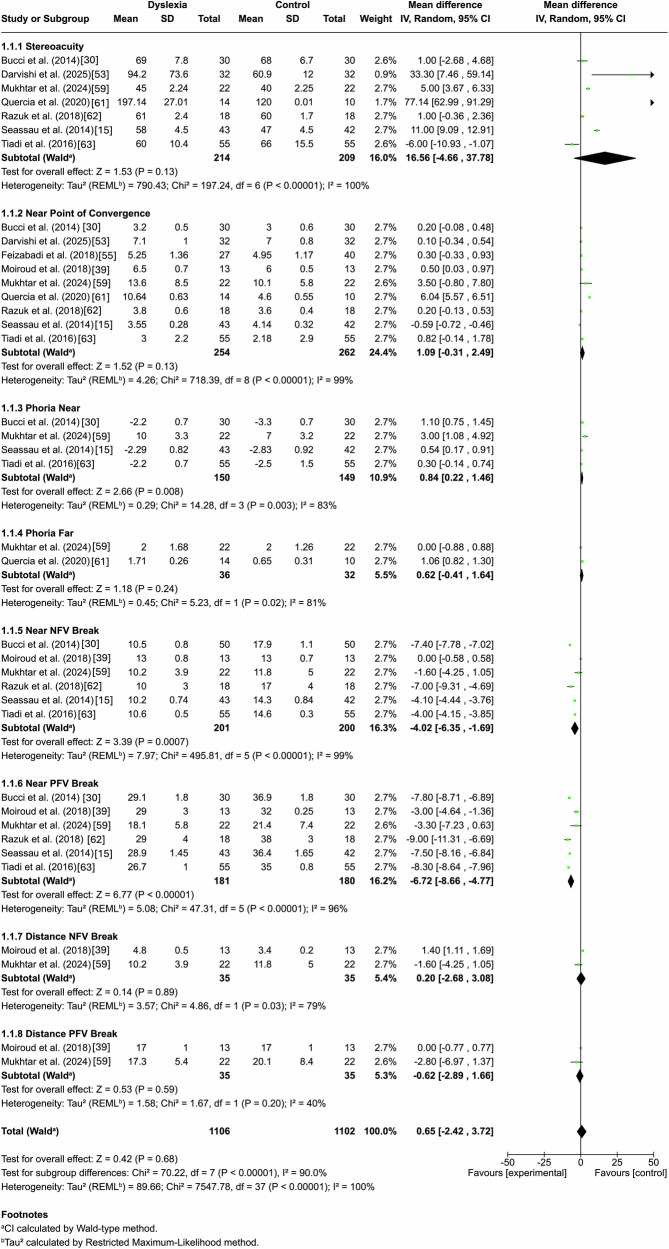
Pooled binocular vision outcomes (stereopsis, heterophoria (phoria), convergence and fusional vergence) in individuals with dyslexia and controls.

Figure 2 presents the pooled results for binocular vision parameters in individuals with dyslexia compared to control groups. Stereopsis was analysed across seven studies [15, 30, 53, 59, 61–63], including 214 participants with dyslexia and 209 controls. The overall mean difference was 16.56 (95% CI: −46.66 to 37.78), indicating no statistically significant difference between groups, with considerable heterogeneity (*I*² = 100%).

For the NPC, nine studies [15, 30, 39, 53, 55, 59, 61–63] with 254 dyslexic and 262 control participants yielded a pooled mean difference of 1.09 (95% CI: −0.31 to 2.49), also not statistically significant and high heterogeneity was observed (*I*² = 99%).

Regarding heterophoria, four studies [15, 30, 59, 63] assessed near heterophoria (150 dyslexic, 149 controls) and two studies [59, 61] assessed far heterophoria (36 dyslexic, 32 controls). The pooled mean difference for near heterophoria was 0.84 (95% CI: 0.22 to 1.46), which was statistically significant and indicated higher exophoria in dyslexic individuals (*I*² = 83%). For far heterophoria, the pooled mean difference was 0.62 (95% CI: −0.41 to 1.64), not reaching statistical significance (*I*² = 81%).

For fusional vergence ranges, six studies [15, 30, 39, 59, 62, 63] evaluated near negative fusional vergence (NFV) break (201 dyslexic, 200 controls), showing a significant pooled mean difference of −4.02 (95% CI: −6.35 to −1.69), indicating reduced NFV in the dyslexia group (*I*² = 99%). Six studies [15, 30, 39, 59, 62, 63] assessed near positive fusional vergence (PFV) break (181 dyslexic, 180 controls), with a pooled mean difference of −6.72 (95% CI: −8.66 to −4.77), again demonstrating significantly reduced PFV among dyslexic individuals (*I*² = 96%). For distance NFV break (two studies [39, 59]) and distance PFV break (two studies [39, 59]), no significant group differences were found, with pooled mean differences of 0.20 (95% CI: −2.68 to 3.08) and −0.62 (95% CI: −2.89 to 1.66), respectively.

Overall, these results indicate that while stereopsis and NPC did not differ significantly between groups, individuals with dyslexia showed significantly greater near exophoria and reduced near fusional vergence ranges (both NFV and PFV) compared to controls. Heterogeneity was substantial for most outcomes, suggesting variability among studies.

**Fig. 3:**
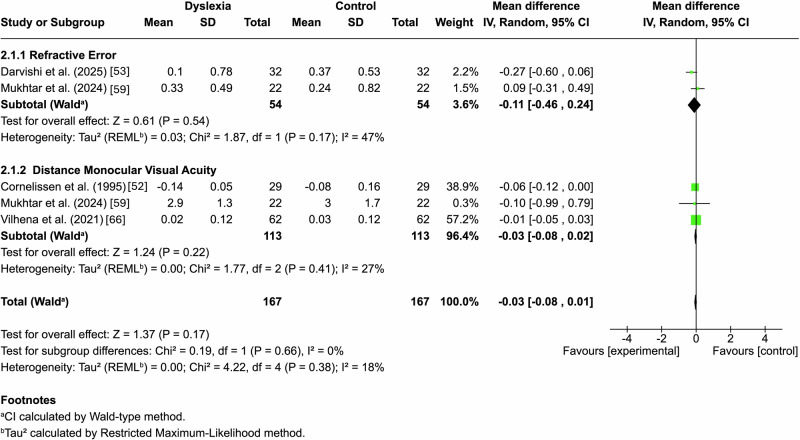
Refractive error and distance visual acuity outcomes in individuals with dyslexia compared to control groups.

Figure 3 presents the pooled results for refractive error and distance monocular visual acuity in individuals with dyslexia compared to control groups. Refractive error was analysed in two studies [53, 59] comprising 54 participants with dyslexia and 54 controls. The pooled mean difference was −0.11 (95% CI: −0.46 to 0.24), indicating no statistically significant difference between groups (*I*² = 47%).

Distance monocular visual acuity was assessed in three studies [52, 59, 66], with a combined sample of 113 participants with dyslexia and 113 controls. The overall mean difference was −0.03 (95% CI: −0.08 to 0.01), also showing no significant difference between groups (*I*² = 27%).

The total pooled analysis, combining both refractive error and visual acuity outcomes (167 participants per group), showed an overall mean difference of −0.03 (95% CI: −0.08 to 0.01), again not reaching statistical significance. Heterogeneity was low across outcomes (*I*² = 18%), indicating consistency among studies.

Overall, these findings suggest that neither refractive error nor distance visual acuity differs significantly between individuals with dyslexia and control groups.

**Fig. 4:**
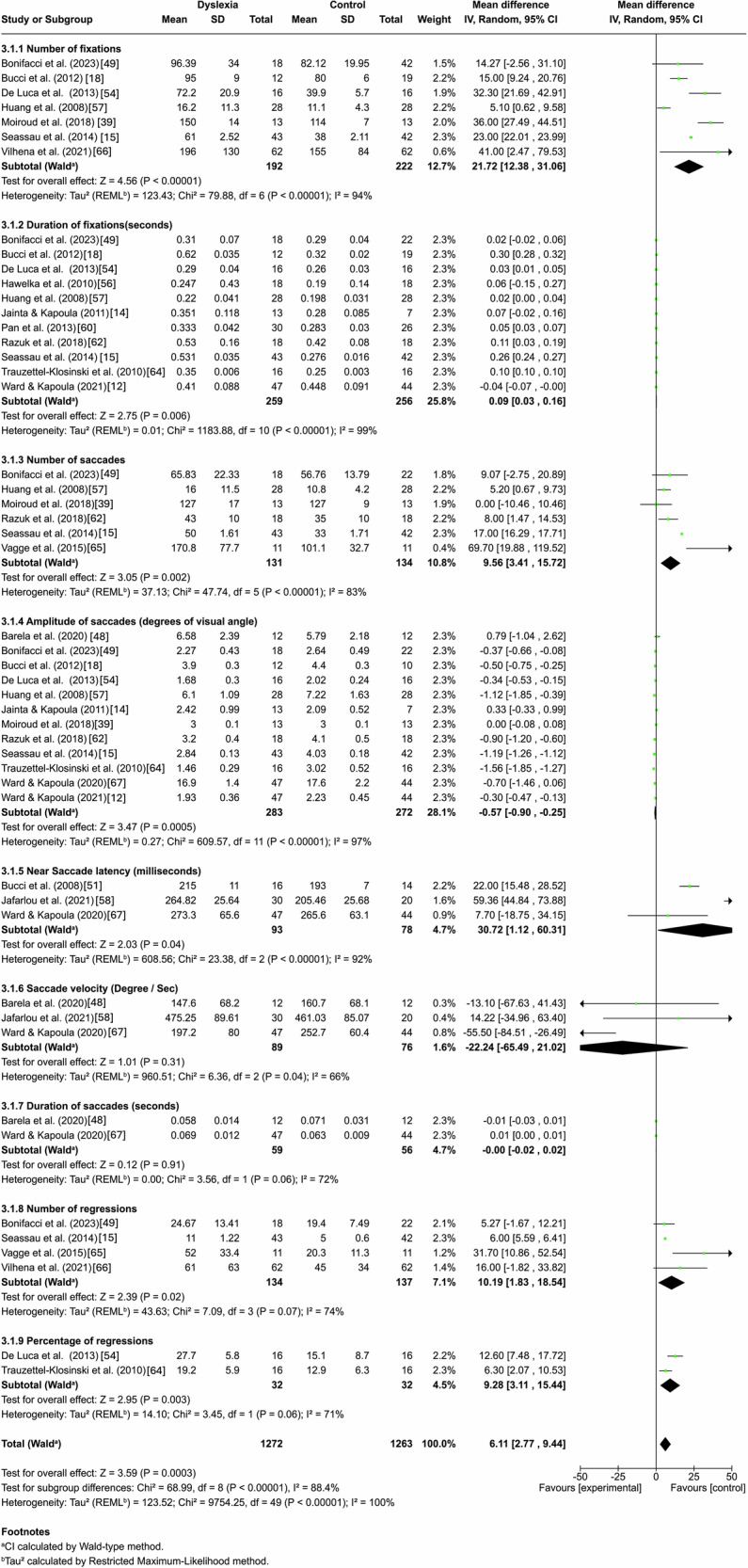
Eye movement outcomes during reading and oculomotor tasks in individuals with dyslexia compared to control groups.

Figure 4 presents the pooled results for eye movement parameters during reading and oculomotor tasks in individuals with dyslexia compared to controls. The number of fixations was assessed in seven studies [15, 18, 39, 49, 54, 57, 66] (192 dyslexic, 222 controls), yielding a significant pooled mean difference of 21.72 (95% CI: 12.38 to 31.06), with higher fixation counts observed in dyslexic participants (*I*² = 94%).

Duration of fixations (in seconds), reported in eleven studies [12, 14, 15, 18, 49, 54, 56, 57, 60, 62, 64] (259 dyslexic, 256 controls), also showed a small but significant increase in the dyslexia group, with a pooled mean difference of 0.09 (95% CI: 0.03 to 0.16; *I*² = 99%).

The number of saccades was analysed in six studies [15, 39, 49, 57, 62, 65] (131 dyslexic, 131 controls), revealing a significant pooled mean difference of 9.56 (95% CI: 3.41 to 15.72; *I*² = 83%), indicating that dyslexic individuals performed more saccades during tasks.

For amplitude of saccades (in degrees of visual angle), twelve studies [12, 14, 15, 18, 39, 48, 49, 54, 57, 62, 64, 67] (283 dyslexic, 272 controls) showed a significant overall reduction in the dyslexia group, with a pooled mean difference of −0.57 (95% CI: −0.90 to −0.25; *I*² = 97%).

Near saccadic latency, analysed in three studies [51, 58, 67] (93 dyslexic, 78 controls), was increased significantly among dyslexic participants, with a mean difference of 30.72 ms (95% CI: 1.12 to 60.31; *I*² = 92%). Saccadic velocity (three studies [48, 58, 67], 89 dyslexic, 86 controls) was reduced significantly in the dyslexia group, with a pooled mean difference of −22.24°/s (95% CI: −45.49 to −1.02; *I*² = 66%). Saccade duration, analysed in two studies [48, 67], showed no significant group difference.

The number of regressions was reported in four studies [15, 49, 65, 66] (134 dyslexic, 137 controls), with a significant pooled mean difference of 10.19 (95% CI: 1.83 to 18.54; *I*² = 74%) favouring higher regression counts in dyslexic participants. Two additional studies [54, 64] found a significantly greater percentage of regressions (mean difference 9.28, 95% CI: 3.11 to 15.44).

Overall, individuals with dyslexia demonstrated altered oculomotor behaviour during reading and visual tasks, characterised by increased fixation counts and durations, higher numbers of saccades and regressions, prolonged saccade latency and reduced saccade amplitude and velocity compared to controls. Although these findings were consistent in direction across most studies, substantial heterogeneity was observed for several parameters, reflecting variability in task demands, eye-tracking methodologies and participant characteristics.

**Fig. 5:**
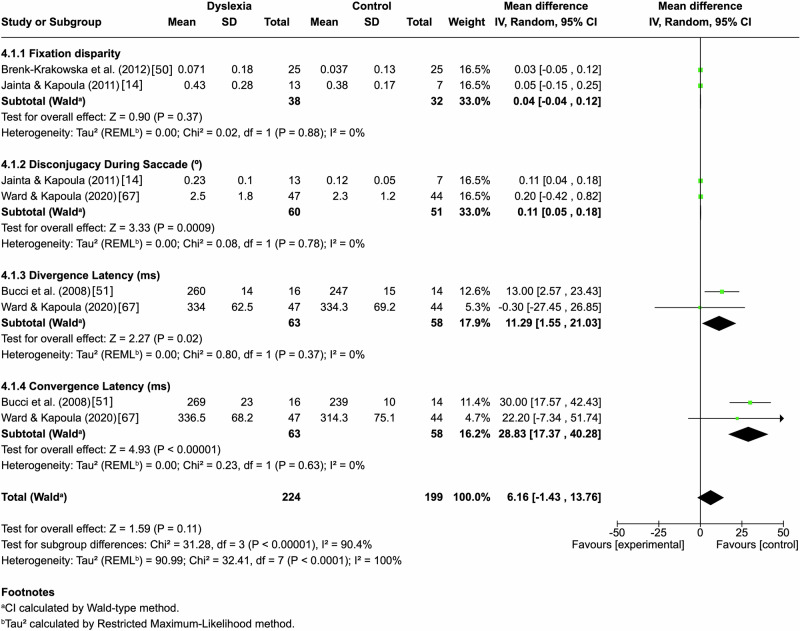
Disparity, disconjugacy and vergence latency outcomes in individuals with dyslexia compared to control groups.

Figure 5 summarises the pooled results for fixation disparity, disconjugacy during saccades and vergence latencies in individuals with dyslexia versus control groups. Fixation disparity was assessed in two studies [14, 50] (38 dyslexic, 32 controls), showing no significant difference between groups (mean difference 0.04, 95% CI: −0.04 to 0.12; *I*² = 0%). Disconjugacy during saccades was evaluated in two studies [14, 67] (60 dyslexic, 51 controls), revealing a small but statistically significant pooled mean difference of 0.11° (95% CI: 0.05 to 0.18; *I*² = 0%), indicating greater disconjugacy in dyslexic participants.

Divergence latency (milliseconds) was reported in two studies [51, 67] (63 dyslexic, 58 controls), with a significant pooled mean difference of 11.29 ms (95% CI: 1.55 to 21.03; *I*² = 0%), indicating increased divergence latency in the dyslexia group. Convergence latency, also from two studies [51, 67] (63 dyslexic, 58 controls), was significantly longer in dyslexic individuals, with a pooled mean difference of 28.83 ms (95% CI: 17.37 to 40.28; *I*² = 0%). The overall pooled analysis across these measures (224 dyslexic, 199 controls) yielded a non-significant mean difference of 6.16 (95% CI: −1.43 to 13.76). Notably, heterogeneity was low (*I*² = 0%) within individual subgroups, but high for the overall pooled effect (*I*² = 100%).

These findings indicate that while fixation disparity did not differ between groups, individuals with dyslexia exhibited significantly greater disconjugacy during saccades and prolonged divergence and convergence latencies compared to controls.

### Sensitivity Analysis

**Fig. 6:**
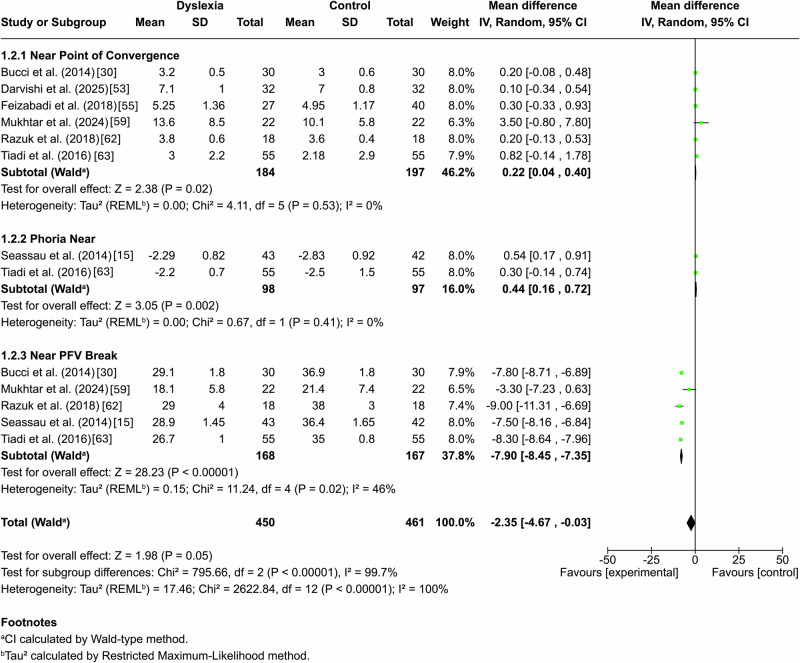
Sensitivity analysis of near point of convergence, heterophoria (phoria) and fusional vergence after exclusion of influential studies.

A sensitivity analysis was conducted by excluding specific studies identified as major contributors to heterogeneity in binocular vision outcomes (Fig. 6). For the NPC, the exclusion of Moiroud et al. [39], Quercia et al. [61], Razuk et al. [62] and Seassau et al. [15] led to a reduced pooled mean difference of 0.22 (95% CI: 0.04 to 0.40), which remained statistically significant. Importantly, this adjustment decreased heterogeneity from 99% to 0%, indicating high consistency across the remaining studies. Similarly, for near heterophoria, the removal of Bucci et al. [30] and Mukhtar et al. [59] reduced the pooled mean difference to 0.44 (95% CI: 0.16 to 0.72) and eliminated heterogeneity (*I*² = 0%). For the near NFV break, exclusion of Mukhtar et al. [59] resulted in a mean difference of −6.35 (95% CI: −7.85 to −4.85) with zero heterogeneity. For the near PFV break, the removal of Moiroud et al. [39] lowered heterogeneity from 99% to 46%, with a pooled mean difference of −8.45 (95% CI: −7.35 to −9.54), confirming the robustness of the finding.

**Fig. 7:**
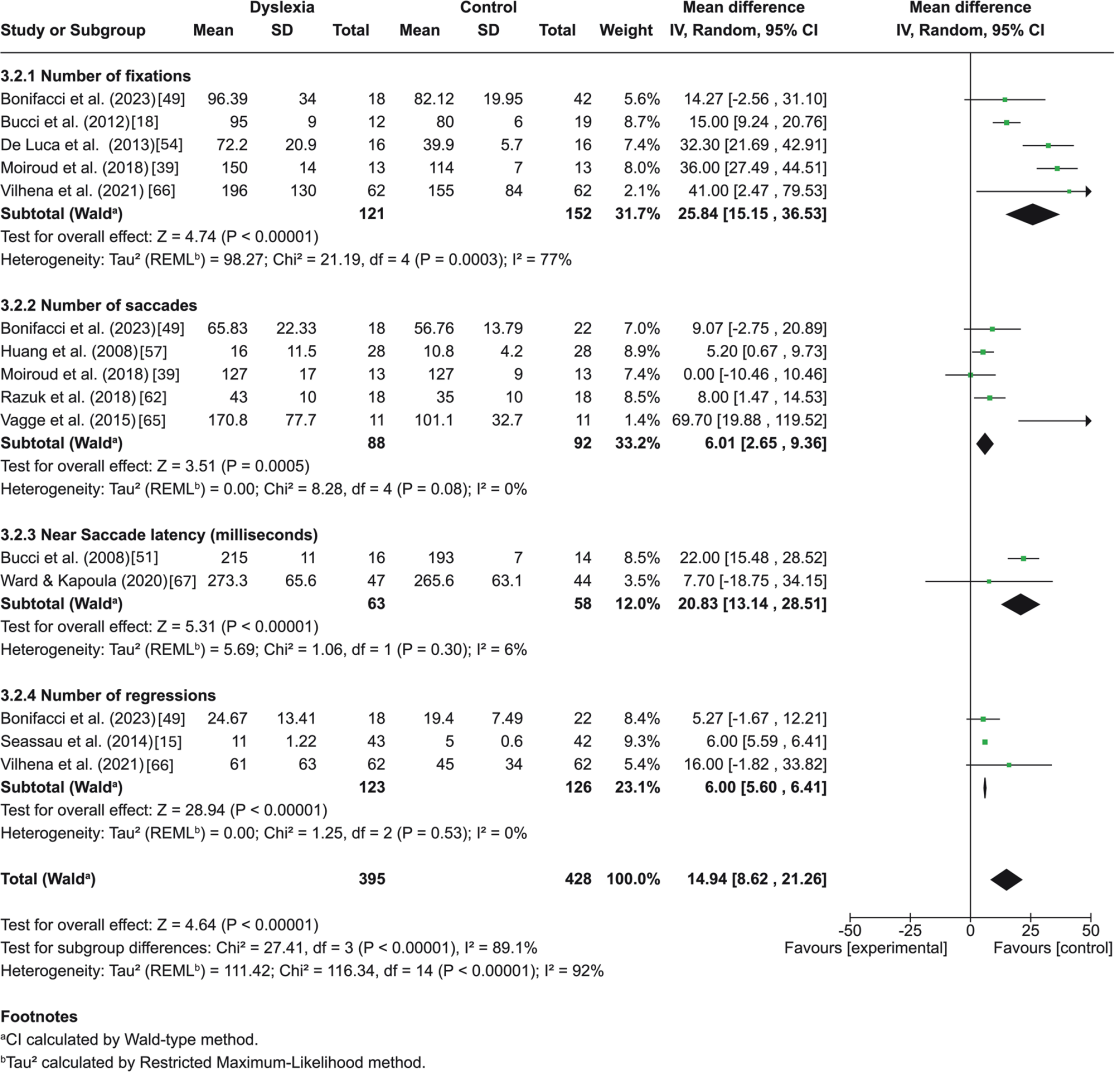
Sensitivity analysis of oculomotor and reading-related eye movement outcomes after exclusion of influential studies.

Another sensitivity analysis was conducted for oculomotor and reading-related eye movement parameters (Fig. 7). For the number of fixations, exclusion of Huang et al. [57] and Seassau et al. [15] resulted in a pooled mean difference of 25.54 (95% CI: 15.15 to 35.93), maintaining a significant difference and substantially reducing heterogeneity from 94% to 77%. For the number of saccades, the removal of Seassau et al. [15], which had a particularly large effect size and wide confidence interval, led to a pooled mean difference of 6.01 (95% CI: 1.65 to 10.36), reducing heterogeneity from 83% to 0%. For near saccadic latency, excluding Jafarlou et al. [58], which contributed to considerable variance, yielded a pooled mean difference of 20.83 ms (95% CI: 13.14 to 28.51), with heterogeneity dropping from 92% to 6%. This demonstrates the effect is robust and consistent across the remaining studies. Regarding the number of regressions, exclusion of Vagge et al. [65] resulted in a pooled mean difference of 6.09 (95% CI: 5.60 to 6.41), and heterogeneity was completely eliminated (*I*² = 0%).

### Publication Bias

**Fig. 8:**
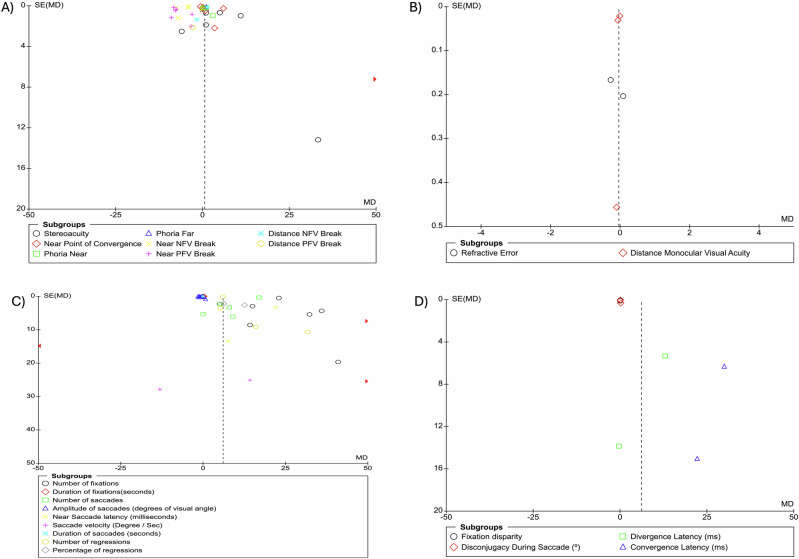
Assessment of publication bias. Funnel plots showing the relationship between effect size (mean difference) and standard error for: **A** Pooled binocular vision outcomes (stereopsis, phoria, convergence, and fusional vergence), **B** Refractive error and distance visual acuity outcomes, **C** Eye movement outcomes during reading and oculomotor tasks and **D** Disparity, disconjugacy, and vergence latency outcomes. Visual inspection suggests possible asymmetry for some outcome groups.

Publication bias was assessed using funnel plots for all main outcome domains in the meta-analysis, including refractive error, distance visual acuity, binocular vision parameters (such as stereoacuity, NPC, heterophoria and fusional vergence) and oculomotor and reading-related eye movement measures (including fixations, saccades, regressions and saccadic parameters). Visual inspection of the funnel plots (Fig. 8) revealed some degree of asymmetry across several outcome groups, particularly for binocular vision, oculomotor function and eye movement outcomes during reading. This asymmetry suggests the possibility of publication bias and/or small-study effects in these domains. The degree of asymmetry varied by parameter, with more pronounced effects observed in outcomes with larger effect sizes and heterogeneity.

### GRADE

The GRADE summary of findings for all key visual and oculomotor outcomes is presented in Table 3. The overall certainty of evidence was rated as very low for binocular vision outcomes and low for visual acuity, refractive error, oculomotor and reading eye movement outcomes, as well as vergence and latency parameters. These downgrades were primarily due to concerns regarding risk of bias, high inconsistency across studies and imprecision in the effect estimates. The presence of substantial heterogeneity and the predominance of non-randomised study designs further limit the certainty of the evidence. Consequently, the interpretation of pooled results should be approached with caution, and there is a clear need for further well-designed, high-quality studies to confirm and expand upon these findings.

**Table 3:** GRADE assessment of the quality of the evidence and the strength of the recommendations.

Certainty assessment							№ of patients		Effect		Certainty	Importance
№ of studies	Study design	Risk of bias	Inconsistency	Indirectness	Imprecision	Other considerations	[intervention]	[comparison]	Relative (95% CI)	Absolute (95% CI)		
Binocular vision outcomes												
9	non-randomised studies	serious	serious^a^	not serious	serious^b^	none	240/486 (49.4%)	245/486 (50.4%)	OR 0.65 (−2.42 to 3.72)	106 fewer per 1000 (from 287 more to 1000 more)	⨁◯◯◯ Very low^a,b^	CRITICAL
Visual acuity and refractive error												
4	non-randomised studies	not serious	not serious	not serious	not serious	none	145/290 (50.0%)	145/290 (50.0%)	OR −0.03 (−0.08 to 0.01)	531 fewer per 1000 (from 587 fewer to 490 fewer)	⨁⨁◯◯ Low	CRITICAL
Oculomotor and reading eye movements												
18	non-randomised studies	not serious	not serious	not serious	not serious	none	450/902 (49.9%)	452/902 (50.1%)	OR 6.11 (2.77 to 9.44)	359 more per 1000 (from 235 more to 403 more)	⨁⨁◯◯ Low	CRITICAL
Vergence and latency outcomes												
4	non-randomised studies	not serious	not serious	not serious	not serious	none	101/191 (52.9%)	90/191 (47.1%)	OR 6.16 (−1.43 to 13.76)	375 more per 1000 (from 453 more to 1000 more)	⨁⨁◯◯ Low	CRITICAL

*CI* confidence interval, *OR* odds ratio.

## Discussion

This study reveals that dyslexic individuals exhibit significant anomalies in oculomotor control and binocular function, while basic optical parameters such as refractive error and visual acuity do not differ meaningfully from those found in typical readers. These results require careful contextualisation in light of previous research.

In the domain of binocular vision, the present meta-analysis reveals that individuals with dyslexia have greater near exophoria and reduced near fusional vergence ranges when compared to controls, a finding that aligns with the results reported by Mukhtar et al. [59] who demonstrated an increased prevalence of accommodative insufficiency and significantly reduced distance PFV recovery in children with dyslexia in a Nigerian cohort, indicating that specific aspects of binocular vision, particularly accommodation and certain fusional reserves, are systematically compromised in this population. This convergence of evidence across diverse geographic and cultural settings points to reduced vergence capacity as a potential core feature associated with dyslexia. However, the current data also show that stereopsis and NPC do not differ significantly between groups, which is consistent with the findings from Feizabadi et al. [55], who also did not observe deficits in these domains among dyslexic children. This similarity across studies implies that not all binocular visual skills are equally affected and that the most sensitive indicators of dysfunction may lie in vergence flexibility rather than static stereoacuity or convergence ability.

Yet, some discrepancies arise when comparing these findings to previous meta-analyses, such as that by Temelturk et al. [68], who reported broader deficits in binocular function, including stereopsis and NPC and argued that binocular anomalies are neither universal nor exclusive to dyslexia, but may overlap with other neurodevelopmental disorders. This difference could reflect heterogeneity in diagnostic protocols, as the studies included here strictly required multidisciplinary or standardised clinical diagnosis, while some previous reviews included broader learning difficulties. These methodological contrasts highlight the importance of precise phenotyping in future work.

The current review identifies robust and consistent oculomotor anomalies in dyslexic readers, encompassing increased fixation counts and durations, more frequent and slower saccades, greater regressions, prolonged saccade latency and both reduced amplitude and velocity during reading and visual tasks. These results closely align with the results of Hawelka et al. [56] and Trauzettel-Klosinski et al. [69], both of whom have documented pronounced oculomotor inefficiency and instability among dyslexic children, as evidenced by increased fixational demands and a higher rate of backward saccades during reading. The similarity in these results across different orthographies and languages reinforces the universality of the oculomotor phenotype in dyslexia. This pattern is further confirmed by Temelturk et al. [68], whose review highlighted the persistence of binocular and oculomotor deficits irrespective of the linguistic context and by Bonifacci et al. [49], who observed these abnormalities in Italian-speaking children with dyslexia as well. The close agreement among these studies and the present meta-analysis, which included data from over ten countries, strengthens the interpretation that oculomotor control deficits serve as a reliable, cross-cultural biomarker of reading difficulties.

Nevertheless, subtle differences in the degree and expression of oculomotor dysfunction appear across studies, and these discrepancies may reflect differences in sample age, diagnostic rigour, comorbidity exclusion and task demands. For example, some studies employing visually guided rather than reading-based tasks, such as those by Bucci et al. [51] and Jafarlou et al. [58], have found that oculomotor anomalies persist even outside of linguistic contexts, supporting the notion that these are not simply epiphenomena of reading impairment but may represent a fundamental neurodevelopmental vulnerability. This similarity is significant because it challenges the classical debate as to whether oculomotor deficits are a cause or a consequence of reading problems; the present findings, corroborated by these studies, argue for an independent role of oculomotor immaturity.

Disconjugacy during saccades and prolonged convergence and divergence latencies also emerged as significant discriminators between dyslexic and control groups in this meta-analysis, echoing the results reported by Ward and Kapoula [12, 67] who observed that increased task complexity exacerbates binocular coordination deficits in dyslexia. This close agreement in both magnitude and direction of effect across studies strengthens the case that the integration of binocular control and oculomotor planning is particularly fragile in individuals with dyslexia. It is worth noting that such disconjugacy is rarely observed in children with other learning difficulties but not dyslexia, suggesting a degree of specificity. Still, the exact neural mechanisms underlying this vulnerability remain a subject of ongoing investigation, as pointed out by Kristjánsson et al. [70] and by recent neuroimaging work demonstrating atypical activation in frontoparietal and cerebellar networks in dyslexic readers [71].

In contrast, and in agreement with the findings of Cornelissen et al. [52] and Pan et al. [60], the present results show no significant differences between dyslexic and control groups in refractive error or monocular visual acuity. This provides further evidence against the hypothesis that uncorrected refractive anomalies are causally implicated in the aetiology of dyslexia. The consistency of these findings is further corroborated by the umbrella review by Olusanya et al. [72], who concluded that neither hyperopia nor astigmatism is associated with a greater risk of dyslexia at the population level. This alignment across multiple independent samples and review methodologies highlights a persistent misconception in both clinical and educational contexts and underscores the need for a comprehensive assessment that goes beyond standard optometric screening. At the same time, the current results agree with recent clinical guidelines that stress the importance of correcting any comorbid refractive error in all children with reading difficulties, even if such correction is not expected to resolve the core manifestations of dyslexia [1, 73].

When considering visual processing and attentional correlates, these findings should be interpreted within the framework of multifactorial models of dyslexia as advocated by Kristjánsson et al. [70], Perry et al. [74] and Chokron et al. [73]. These models posit that visual and oculomotor vulnerabilities, while not sufficient or necessary for the diagnosis of dyslexia, interact with phonological, attentional and motor control factors to shape the severity and phenotype of reading impairment. The present demonstration of robust group-level differences in oculomotor and binocular domains, in the absence of systematic deficits in acuity or stereopsis, is thus consistent with a risk-modulation model rather than a single-cause theory. This perspective is supported by Soheili-Nezhad et al. [71], whose genetic and imaging data reveal associations between polygenic risk for dyslexia and altered visual-motor brain networks, as well as by El Hmimdi et al. [75], who demonstrated that oculomotor metrics extracted during free-viewing can be used to identify children with dyslexia reliably using machine learning approaches. The similarity in results across neuroimaging, behavioural and computational approaches strengthens the generalisability of this multifactorial conceptualisation.

Where the present results diverge from previous literature, particularly in regard to the magnitude of differences observed in certain binocular vision measures and the limited number of studies reporting contrast sensitivity or other low-level visual metrics, these differences may be attributed to methodological stringency in the inclusion criteria. By restricting analysis to studies with robust case-control designs, standardised diagnostic criteria and comprehensive statistical reporting, earlier research with less stringent methodologies may have been excluded. Additionally, geographic and demographic variation among samples may contribute to heterogeneity, as highlighted by Vilhena et al. [66], who observed cultural differences in oculomotor task performance between Brazilian and Portuguese children, although the general trend of dyslexic oculomotor inefficiency persisted in both groups.

Substantial heterogeneity in effect sizes across studies also reflects the diversity of experimental paradigms employed, ranging from natural reading to rapid automatised naming or visual search and from laboratory-based eye tracking to clinical orthoptic testing. This variability is a recognised limitation in the literature and underscores the need for greater methodological standardisation, as called for by both Perry et al. [74] and Kristjánsson et al. [70]. The sensitivity analyses used here, in which the removal of outlier studies reduced heterogeneity and did not eliminate the main effects, provide reassurance regarding the robustness of the principal conclusions but do not fully address the problem of cross-study comparability.

Despite these challenges, the present findings consistently reinforce the notion that oculomotor control and binocular function are integral to the visual phenotype of dyslexia and should be assessed systematically in multidisciplinary diagnostic protocols. At the same time, these results highlight the importance of differential diagnosis, since not all children with reading problems display these anomalies and since similar profiles may be found, albeit less frequently, in other neurodevelopmental disorders such as attention deficit hyperactivity disorder (ADHD) and cerebral visual impairment [61, 73, 76].

In terms of strengths, this review benefits from a comprehensive protocolised approach, strict adherence to PRISMA and AMSTAR-2 standards, independent risk of bias assessment using the MINORS tool and the integration of a geographically diverse sample encompassing studies from Europe, Asia, South America and Africa. Dual data extraction and rigorous sensitivity analyses further bolster the reliability and transparency of the results. Moreover, the application of the GRADE framework enables clear communication of evidence certainty, distinguishing outcomes supported by robust data from those limited by inconsistency or imprecision. Nonetheless, some limitations must be acknowledged. The observational, non-randomised nature of all included studies precludes causal inference and despite attempts to minimise heterogeneity through strict inclusion criteria and sensitivity analysis, considerable variability in diagnostic definitions, measurement protocols and participant age remains. Additionally, some potentially relevant studies, particularly older publications, could not be included in the quantitative synthesis due to insufficient reporting of summary statistics required for meta-analysis (e.g., means and standard deviations), rather than lack of access to original datasets. This reflects historical reporting practices, when data sharing and standardised statistical reporting were not routinely required and may have influenced study selection and pooled estimates. In addition, commonly used performance-based or screening tools in clinical practice were not included in the quantitative synthesis, as they do not provide direct, standardised visual or oculomotor parameters suitable for meta-analytic pooling. The potential for publication bias, suggested by funnel plot asymmetry in binocular and oculomotor domains, may have resulted in an overrepresentation of studies finding significant differences, a risk not fully mitigated despite exhaustive search and inclusion of gray literature. Furthermore, the lack of randomised controlled trials evaluating whether correction of binocular or oculomotor anomalies improves reading performance limits the translational value of these findings for clinical practice. Lastly, the underrepresentation of studies from low- and middle-income countries also constrains generalisability. Additionally, the exclusion of studies that assessed broader learning disability populations or relied on non-standardised or non-comparable outcome measures may have reduced the scope of the quantitative synthesis, although it increased internal validity and comparability across included studies.

Looking ahead, the field would benefit from greater standardisation of both diagnostic and measurement protocols, as well as from prospective longitudinal studies tracking the developmental trajectory of visual and oculomotor anomalies in dyslexia from early childhood through adolescence. Randomised controlled trials evaluating the impact of targeted interventions (whether orthoptic, cognitive or multimodal) on both visual function and reading outcomes are urgently needed to translate these findings into actionable strategies. There is also a need for research in underrepresented populations and for the development and validation of integrated screening tools that combine oculomotor, binocular and cognitive measures for improved diagnosis and intervention planning. Neuroimaging and genetic studies, such as those by Soheili-Nezhad et al. [71], offer promising avenues for elucidating the mechanistic underpinnings of these phenotypes and may, in the future, enable personalised intervention strategies.

## Conclusions

This systematic review and meta-analysis revealed that individuals with dyslexia consistently exhibit anomalies in oculomotor control and binocular vision, such as increased near exophoria, reduced fusional vergence and more frequent oculomotor inefficiency, despite showing no consistent differences in refractive error or visual acuity compared to typical readers. These findings suggest that standard vision care assessments in dyslexic children should be complemented by targeted evaluation of binocular and oculomotor functions. While such anomalies may not be the primary cause of dyslexia, their presence can contribute to increased visual effort and reading challenges. Incorporating these assessments into multidisciplinary diagnostic protocols could enhance the detection and management of visual factors affecting reading. However, the substantial heterogeneity among studies and the lack of randomised trials limit the strength of recommendations. Further longitudinal and interventional research is needed to clarify the clinical significance of these visual dysfunctions and to establish evidence-based strategies for intervention.

## Supplementary Information

Additional File 1

Additional File 2

## Data Availability

All data generated or analysed during this study are included in this published article and its supplementary information files.

## References

[1] Bilbao C, Piñero DP. Diagnosis of oculomotor anomalies in children with learning disorders. Clin Exp Optom. 2020;103:597–609. 10.1111/cxo.13024. 31869866 10.1111/cxo.13024

[2] Zhao M, Gersch TM, Schnitzer BS, Dosher BA, Kowler E. Eye movements and attention: the role of pre-saccadic shifts of attention in perception, memory and the control of saccades. Vision Res. 2012;74:40–60. 10.1016/j.visres.2012.06.017. 22809798 10.1016/j.visres.2012.06.017 PMC3623695

[3] Pratt J, Trottier L. Pro-saccades and anti-saccades to onset and offset targets. Vision Res. 2005;45:765–74. 10.1016/j.visres.2004.05.019. 15639503 10.1016/j.visres.2004.05.019

[4] Blais C, Fiset D, Arguin M, Jolicoeur P, Bub D, Gosselin F. Reading between eye saccades. PLoS ONE. 2009;4:e6448 10.1371/journal.pone.0006448. 19649292 10.1371/journal.pone.0006448 PMC2714180

[5] Barbiero C, Lonciari I, Montico M, Monasta L, Penge R, Vio C, et al. The submerged dyslexia iceberg: how many school children are not diagnosed?. PLoS ONE. 2012;7:e48082 10.1371/journal.pone.0048082. 23118930 10.1371/journal.pone.0048082 PMC3485303

[6] Barbiero C, Montico M, Lonciari I, Monasta L, Penge R, Vio C, et al. The lost children: the underdiagnosis of dyslexia in Italy. PLoS ONE. 2019;14:e0210448 10.1371/journal.pone.0210448. 30673720 10.1371/journal.pone.0210448 PMC6343900

[7] Wagner RK, Zirps FA, Edwards AA, Wood SG, Joyner RE, Becker BJ, et al. The prevalence of dyslexia: a new approach to its estimation. J Learn Disabil. 2020;53:354–65. 10.1177/0022219420920377. 32452713 10.1177/0022219420920377 PMC8183124

[8] Rajesh R, Sunney H. Assessment on prevalence and risk factors of dyslexia among primary school students. Indian J Psychiatr Nurs. 2021;18:85–9. 10.4103/iopn.iopn_18_21.

[9] Vikesdal GH, Mon-Williams M, Langaas T. Optometric disorders in children and adults with dyslexia. Scand J Educ Res. 2020;64:601–11. 10.1080/00313831.2019.1595715.

[10] Protopapas A, Parrila R. Is dyslexia a brain disorder?. Brain Sci. 2018;8:61 10.3390/brainsci8040061. 29621138 10.3390/brainsci8040061 PMC5924397

[11] Raghuram A, Gowrisankaran S, Swanson E, Zurakowski D, Hunter DG, Waber DP. Frequency of visual deficits in children with developmental dyslexia. JAMA Ophthalmol. 2018;136:1089–95. 10.1001/jamaophthalmol.2018.2797. 30027208 10.1001/jamaophthalmol.2018.2797 PMC6583865

[12] Ward LM, Kapoula Z. Dyslexics’ fragile oculomotor control is further destabilized by increased text difficulty. Brain Sci. 2021;11:11 10.3390/brainsci11080990. 10.3390/brainsci11080990 PMC8394394 34439612

[13] Danelli L, Berlingeri M, Bottini G, Ferri F, Vacchi L, Sberna M, et al. Neural intersections of the phonological, visual magnocellular and motor/cerebellar systems in normal readers. Hum Brain Mapp. 2013;34:2669–87. 10.1002/hbm.22098. 22736513 10.1002/hbm.22098 PMC6870382

[14] Jainta S, Kapoula Z. Dyslexic children are confronted with unstable binocular fixation while reading. PLoS ONE. 2011;6:e18694. 10.1371/journal.pone.0018694. 10.1371/journal.pone.0018694 PMC3071843 21494641

[15] Seassau M, Gérard CL, Bui-Quoc E, Bucci MP. Binocular saccade coordination in reading and visual search. Front Integr Neurosci. 2014;8. 10.3389/fnint.2014.00085. 10.3389/fnint.2014.00085 PMC4214188 25400559

[16] Bucci MP, Brémond-Gignac D, Kapoula Z. Poor binocular coordination of saccades in dyslexic children. Graefes Arch Clin Exp Ophthalmol. 2008;246:417–28. 10.1007/s00417-007-0723-1. 18046570 10.1007/s00417-007-0723-1

[17] Razuk M, Peyre H, Gerard C, Bucci M. Eye movements and postural control in dyslexic children. PLoS ONE. 2018;13:e0198001 10.1371/journal.pone.0198001. 29795687 10.1371/journal.pone.0198001 PMC5967793

[18] Bucci MP, Nassibi N, Gerard CL, Bui-Quoc E, Seassau M. Immaturity of the oculomotor saccade and vergence interaction in dyslexic children. PLoS ONE. 2012;7:7 10.1371/journal.pone.0033458. 10.1371/journal.pone.0033458 PMC3306409 22438934

[19] Yang Q, Bucci MP, Kapoula Z. The latency of saccades, vergence, and combined eye movements. Invest Ophthalmol Vis Sci. 2002;43:2939–49. 12202513

[20] Daniel F, Kapoula Z. Induced vergence-accommodation conflict reduces cognitive performance. Sci Rep. 2019;9:1247. 10.1038/s41598-018-37778-y. 30718625 10.1038/s41598-018-37778-y PMC6361994

[21] Stein J. What is developmental dyslexia?. Brain Sci. 2018;8:26 10.3390/brainsci8020026. 29401712 10.3390/brainsci8020026 PMC5836045

[22] Gori S, Cecchini P, Bigoni A, Molteni M, Facoetti A. Magnocellular-dorsal pathway and sub-lexical route in developmental dyslexia. Front Hum Neurosci. 2014;8:8 10.3389/fnhum.2014.00460. 25009484 10.3389/fnhum.2014.00460 PMC4068287

[23] Gori S, Seitz AR, Ronconi L, Franceschini S, Facoetti A. Multiple causal links between magnocellular–dorsal pathway deficit and developmental dyslexia. Cereb Cortex. 2016;26:4356–69. 10.1093/cercor/bhv206. 26400914 10.1093/cercor/bhv206 PMC6317503

[24] Stein J. The current status of the magnocellular theory of developmental dyslexia. Neuropsychologia. 2019;130:66–77. 10.1016/j.neuropsychologia. 2018.03.022. 29588226 10.1016/j.neuropsychologia.2018.03.022

[25] Olulade OA, Napoliello EM, Eden GF. Abnormal visual motion processing is not a cause of dyslexia. Neuron. 2013;79:180–90. 10.1016/j.neuron.2013.05.002. 23746630 10.1016/j.neuron.2013.05.002 PMC3713164

[26] Dusek W, Pierscionek BK, McClelland JF. A survey of visual function in an Austrian population of school-age children with reading and writing difficulties. BMC Ophthalmol. 2010;10:16. 10.1186/1471-2415-10-16. 20500851 10.1186/1471-2415-10-16 PMC2887790

[27] Wahlberg Ramsay M, Nordström M, Salkic J, Brautaset R. Evaluation of aspects of binocular vision in children with dyslexia. Strabismus. 2012;20:139–44. 10.3109/09273972.2012.735335. 23211137 10.3109/09273972.2012.735335

[28] Kapoula Z, Bucci MP, Jurion F, Ayoun J, Afkhami F, Brémond-Gignac D. Evidence for frequent divergence impairment in French dyslexic children. Graefes Arch Clin Exp Ophthalmol. 2007;245:931–6. 10.1007/s00417-006-0490-4. 17186259 10.1007/s00417-006-0490-4

[29] Borsting E, Rouse MW, Deland PN, Hovett S, Kimura D, Park M, Stephens B. Association of symptoms and convergence and accommodative insufficiency in schoolage children. Optometry. 2003;74:25–34. 12539890

[30] Bucci MP, Mélithe D, Ajrezo L, Bui-Quoc E, Gérard CL. The influence of oculomotor tasks on postural control in dyslexic children. Front Hum Neurosci. 2014;8:8 10.3389/fnhum.2014.00981. 25538603 10.3389/fnhum.2014.00981 PMC4260515

[31] VIP-HIP Study G, Kulp MT, Ciner E, Maguire M, Moore B, Pentimonti J, et al. Uncorrected hyperopia and preschool early literacy. Ophthalmology. 2016;123:123–9. 10.1016/j.ophtha.2015.11.023. 10.1016/j.ophtha.2015.11.023 PMC4808323 26826748

[32] Evans BJW, Drasdo N, Richards IL. An investigation of some sensory and refractive visual factors in dyslexia. Vision Res. 1994;34:1913–26. 10.1016/0042-6989(94)90315-8. 7941393 10.1016/0042-6989(94)90315-8

[33] Fernandez-Velazquez F, Fernandez-Fidalgo M. Do DEM test scores change with respect to the language?. Optom Vis Sci. 1996;72:902–6. 10.1097/00006324-199512000-00009. 10.1097/00006324-199512000-00009 8749338

[34] Webber A, Wood J, Gole G, Brown B. DEM test, Visagraph eye movement recordings, and reading ability in children. Optom Vis Sci. 2011;88:295–302. 10.1097/OPX.0b013e31820846c0. 21217407 10.1097/OPX.0b013e31820846c0

[35] Garzia RP, Richman JE, Nicholson SB, Gaines CS. A new visual-verbal saccade test: the developmental eye movement test (DEM). J Am Optom Assoc. 1990;61:124–35. 2313029

[36] Facchin A. Spotlight on the developmental eye movement (DEM) test. Clin Optom. 2021;13:73–81. 10.2147/OPTO.S232487. 10.2147/OPTO.S232487 PMC7936383 33688290

[37] Alvarez C, Puell M. Relationship between oculomotor scanning determined by the DEM test and a contextual reading test. Graefes Arch Clin Exp Ophthalmol. 2009;247:1243–9. 10.1007/s00417-009-1076-8. 19347678 10.1007/s00417-009-1076-8

[38] Ayton L, Abel L, Fricke T, McBrien N. Developmental eye movement test: what is it really measuring?. Optom Vis Sci. 2009;86:722–30. 10.1097/OPX.0b013e3181a6a4b3. 19417709 10.1097/OPX.0b013e3181a6a4b3

[39] Moiroud L, Gerard CL, Peyre H, Bucci MP. Developmental eye movement test and dyslexic children. PLoS ONE. 2018;13:13 10.1371/journal.pone.0200907. 10.1371/journal.pone.0200907 PMC6128452 30192750

[40] American Academy of Pediatrics, American Association for Pediatric Ophthalmology and Strabismus, American Association of Certified Orthoptists. Joint statement: learning disabilities, dyslexia, and vision. Pediatrics. 2009;124:837–44. 10.1542/peds.2009-1445. 19651597 10.1542/peds.2009-1445

[41] Buzzelli AR. Stereopsis, accommodative and vergence facility: do they relate to dyslexia?. Optom Vis Sci. 1991;68:842–6. 10.1097/00006324-199111000-00002. 1766644 10.1097/00006324-199111000-00002

[42] Evans BJW, Drasdo N, Richards IL. Investigation of accommodative and binocular function in dyslexia. Ophthalmic Physiol Opt. 1994;14:5–19. 10.1111/j.1475-1313.1994.tb00550.x. 8152821 10.1111/j.1475-1313.1994.tb00550.x

[43] Page MJ, McKenzie JE, Bossuyt PM, Boutron I, Hoffmann TC, Mulrow CD, et al. The PRISMA 2020 statement. BMJ. 2021;372:n71 10.1136/bmj.n71. 33782057 10.1136/bmj.n71 PMC8005924

[44] Shea BJ, Reeves BC, Wells G, Thuku M, Hamel C, Moran J, et al. AMSTAR 2. BMJ. 2017;358:j4008 10.1136/bmj.j4008. 28935701 10.1136/bmj.j4008 PMC5833365

[45] Slim K, Nini E, Forestier D, Kwiatkowski F, Panis Y, Chipponi J. Methodological index for non-randomized studies (MINORS). ANZ J Surg. 2003;73:712–6. 10.1046/j.1445-2197.2003.02748.x. 12956787 10.1046/j.1445-2197.2003.02748.x

[46] Higgins JPT, Thomas J, Chandler J, Cumpston M, Li T, Page MJ, et al. Cochrane handbook for systematic reviews of interventions. Hoboken, New Jersey: Wiley-Blackwell; 2019.

[47] Guyatt GH, Thorlund K, Oxman AD, Walter SD, Patrick D, Furukawa TA, et al. GRADE guidelines: preparing summary of findings tables. J Clin Epidemiol. 2013;66:173–83. 10.1016/j.jclinepi.2012.08.001. 23116689 10.1016/j.jclinepi.2012.08.001

[48] Barela JA, Tesima N, Amaral V, Figueiredo GA, Barela A. Visually guided eye movements reduce postural sway in dyslexic children. Neurosci Lett. 2020;725:725 10.1016/j.neulet.2020.134890. 10.1016/j.neulet.2020.134890 32145309

[49] Bonifacci P, Tobia V, Sansavini A, Guarini A. Eye-movements in a text reading task. Brain Sci. 2023;13:13 10.3390/brainsci13030425. 10.3390/brainsci13030425 PMC10046295 36979235

[50] Brenk-Krakowska A, Szady M, Naskrecki R. Fixation disparity curve in dyslexic adults. Opt Appl. 2012;42:805–20.

[51] Bucci MP, Brémond-Gignac D, Kapoula Z. Latency of saccades and vergence eye movements in dyslexic children. Exp Brain Res. 2008;188:1–12. 10.1007/s00221-008-1345-5. 18357443 10.1007/s00221-008-1345-5

[52] Cornelissen P, Richardson A, Mason A, Fowler S, Stein J. Contrast sensitivity and coherent motion detection in dyslexics and controls. Vision Res. 1995;35:1483–94. 10.1016/0042-6989(95)98728-R. 7645277 10.1016/0042-6989(95)98728-r

[53] Darvishi A, Sangsefidi N, Shandiz JH, Rad DS, Narooie-Noori F, Khorrami-Nejad M. Visual function deficits in dyslexic children. BMC Ophthalmol. 2025;25:144. 10.1186/s12886-025-03959-3. 40102786 10.1186/s12886-025-03959-3 PMC11917054

[54] De Luca M, Pontillo M, Primativo S, Spinelli D, Zoccolotti P. The eye-voice lead during oral reading in developmental dyslexia. Front Hum Neurosci. 2013;7:696 10.3389/fnhum.2013.00696. 24223541 10.3389/fnhum.2013.00696 PMC3818695

[55] Feizabadi M, Jafarzadehpur E, Akrami M. Accommodation, convergence, and stereopsis in dyslexic schoolchildren. Middle East Afr J Ophthalmol. 2018;25:14–18. 10.4103/meajo.MEAJO_71_17. 29899645 10.4103/meajo.MEAJO_71_17 PMC5974812

[56] Hawelka S, Gagl B, Wimmer H. A dual-route perspective on eye movements of dyslexic readers. Cognition. 2010;115:367–79. 10.1016/j.cognition.2009.11.004. 20227686 10.1016/j.cognition.2009.11.004 PMC2976468

[57] Huang X, Jing J, Zou X-B, Wang M-L, Li X-H, Lin A-H. Eye movement characteristics of Chinese dyslexic children. Chin Med J. 2008;121:1617–21. 19024086

[58] Jafarlou F, Ahadi M, Jarollahi F. Eye movement patterns in Iranian dyslexic children. Auris Nasus Larynx. 2021;48:594–600. 10.1016/j.anl.2020.11.012. 33261980 10.1016/j.anl.2020.11.012

[59] Mukhtar IS, Ezinne NE, Mohamad Shahimin M, Mohd-Ali B, Oghre E, Zeried FM, et al. Binocular vision anomalies among children with dyslexia. Pediatr Rep. 2024;16:566–78. 10.3390/pediatric16030048. 39051235 10.3390/pediatric16030048 PMC11270174

[60] Pan J, Yan M, Laubrock J, Shu H, Kliegl R. Eye-voice span during rapid automatized naming. Dev Sci. 2013;16:967–79. 10.1111/desc.12075. 24118721 10.1111/desc.12075

[61] Quercia P, Pozzo T, Marino A, Guillemant AL, Cappe C, Gueugneau N. Altered cross-modal processing linked to binocular fusion. Clin Ophthalmol. 2020;14:437–48. 10.2147/OPTH.S226690. 32103890 10.2147/OPTH.S226690 PMC7025670

[62] Razuk M, Perrin-Fievez F, Gerard CL, Peyre H, Barela JA, Bucci MP. Effect of colored filters on reading capabilities. Res Dev Disabil. 2018;83:1–7. 10.1016/j.ridd.2018.07.006. 30048864 10.1016/j.ridd.2018.07.006

[63] Tiadi A, Gérard CL, Peyre H, Bui-Quoc E, Bucci MP. Immaturity of visual fixations in dyslexic children. Front Hum Neurosci. 2016;10:58 10.3389/fnhum.2016.00058. 26924975 10.3389/fnhum.2016.00058 PMC4756100

[64] Trauzettel-Klosinski S, Koitzsch AM, Dürrwächter U, Sokolov AN, Reinhard J, Klosinski G. Eye movements in German-speaking children with and without dyslexia. Acta Ophthalmol. 2010;88:681–91. 10.1111/j.1755-3768.2009.01523.x. 19508458 10.1111/j.1755-3768.2009.01523.x

[65] Vagge A, Cavanna M, Traverso CE, Iester M. Evaluation of ocular movements in patients with dyslexia. Ann Dyslexia. 2015;65:24–32. 10.1007/s11881-015-0098-7. 25804764 10.1007/s11881-015-0098-7

[66] de Araújo Vilhena D, Guimarães MR, Guimarães RQ, Pinheiro ÂMV. Magnocellular visual function in developmental dyslexia. Arq Bras Oftalmol. 2021;84:442–8. 34320103 10.5935/0004-2749.20210069 PMC11878433

[67] Ward LM, Kapoula Z. Differential diagnosis of vergence and saccade disorders in dyslexia. Sci Rep. 2020;10:22116. 10.1038/s41598-020-79089-1. 33335200 10.1038/s41598-020-79089-1 PMC7747706

[68] Temelturk RD, Ozer E. Binocular coordination in linguistic and non-linguistic tasks. Ann Dyslexia. 2022;72:426–44. 10.1007/s11881-022-00256-2. 35486327 10.1007/s11881-022-00256-2

[69] Trauzettel-Klosinski S, Faisst T, Schick V, Righetti G, Braun C, Cordey-Henke A, et al. Eye movements in alphabetic and logographic tasks. Sci Rep. 2024;14:28796. 39567570 10.1038/s41598-024-78894-2 PMC11579334

[70] Kristjansson A, Sigurdardottir H. The role of visual factors in dyslexia. J Cogn. 2023;6:31. 37397349 10.5334/joc.287 PMC10312247

[71] Soheili-Nezhad S, Schijven D, Mars RB, Fisher SE, Francks C. Distinct impact modes of polygenic disposition to dyslexia in the adult brain. Sci Adv. 2024;10::eadq2754 10.1126/sciadv.adq2754. 39693421 10.1126/sciadv.adq2754 PMC11654687

[72] Olusanya BO, Smythe T, Ogbo FA, Nair M, Scher M, Davis AC. Global prevalence of developmental disabilities. Front Public Health. 2023;11:1122009 10.3389/fpubh.2023.1122009. 36891340 10.3389/fpubh.2023.1122009 PMC9987263

[73] Chokron S, Dutton GN. From vision to cognition. J Neural Transm. 2023;130:409–24. 10.1007/s00702-022-02572-8. 36547695 10.1007/s00702-022-02572-8

[74] Perry C, Long H. Visual attention in reading and dyslexia. Brain Sci. 2022;12:87 10.3390/brainsci12010087. 35053830 10.3390/brainsci12010087 PMC8773944

[75] El Hmimdi AE, Ward LM, Palpanas T, Sainte Fare Garnot V, Kapoula Z. Predicting dyslexia from eye movements. Brain Sci. 2022;12:1031 10.3390/brainsci12081031. 36009094 10.3390/brainsci12081031 PMC9405842

[76] Hokken MJ, Krabbendam E, van der Zee YJ, Kooiker MJG. Visual selective attention in CVI, ADHD and dyslexia. Child Neuropsychol. 2023;29:357–90. 10.1080/09297049.2022.2057940. 35440276 10.1080/09297049.2022.2057940

