# Supplementary material for: Visual and Oculomotor Function in Developmental Dyslexia: A Systematic Review and Meta-Analysis
Source: Ophthalmic Physiol Opt. 2026 Mar 9;46(2):152–69. doi: 10.1007/s44402-026-00044-0 (PMC13369651; doi:10.1007/s44402-026-00044-0)
Supplement: Supplementary file 2 — Additional File 2 [file 44402_2026_44_MOESM2_ESM.docx]

**Additional File 2.** Full electronic search strategies

Pubmed

Search: **("dyslexia"[Title/Abstract] OR "dyslexic"[Title/Abstract]) AND ("saccadic movement"[Title/Abstract] OR "saccades"[Title/Abstract] OR "stereopsis"[Title/Abstract] OR "ocular motility"[Title/Abstract] OR "vergence dysfunction"[Title/Abstract] OR "oculomotor"[Title/Abstract] OR "oculomotor dysfunction"[Title/Abstract] OR "contrast sensitivity"[Title/Abstract] OR "refractive errors"[Title/Abstract] OR "binocular vision"[Title/Abstract] OR "convergence insufficiency"[Title/Abstract] OR "accommodation disorder"[Title/Abstract])** Sort by: **Publication Date**

("dyslexia"[Title/Abstract] OR "dyslexic"[Title/Abstract]) AND ("saccadic movement"[Title/Abstract] OR "saccades"[Title/Abstract] OR "stereopsis"[Title/Abstract] OR "ocular motility"[Title/Abstract] OR "vergence dysfunction"[Title/Abstract] OR "oculomotor"[Title/Abstract] OR "oculomotor dysfunction"[Title/Abstract] OR "contrast sensitivity"[Title/Abstract] OR "refractive errors"[Title/Abstract] OR "binocular vision"[Title/Abstract] OR "convergence insufficiency"[Title/Abstract] OR "accommodation disorder"[Title/Abstract])

Web of Science

TS=("dyslexia" OR "dyslexic")

AND

TS=("saccadic movement" OR "saccades" OR "stereopsis" OR "ocular motility" OR "vergence dysfunction" OR "oculomotor" OR "oculomotor dysfunction" OR "contrast sensitivity" OR "refractive errors" OR "binocular vision" OR "convergence insufficiency" OR "accommodation disorder")

Scopus

TITLE-ABS-KEY("dyslexia" OR "dyslexic") AND TITLE-ABS-KEY("saccadic movement" OR "saccades" OR "stereopsis" OR "ocular motility" OR "vergence dysfunction" OR "oculomotor" OR "oculomotor dysfunction" OR "contrast sensitivity" OR "refractive errors" OR "binocular vision" OR "convergence insufficiency" OR "accommodation disorder")
